# OA16.02. Longitudinal collection of patient-related outcomes in integrative medicine clinics: a pilot study from the BraveNet Practice Based Research Network

**DOI:** 10.1186/1472-6882-12-S1-O63

**Published:** 2012-06-12

**Authors:** J Dusek, D Abrams

**Affiliations:** 1 Penny George Inst. for Health & Healing, ANW, Allina Health, Minneapolis, USA

## Purpose

To understand the impact of integrative medicine (IM) therapies on patients receiving care at BraveNet practice-based research network (PBRN) via longitudinal collection of patient-reported outcomes. Previously, the BraveNet PBRN conducted a cross-sectional study including over 4,000 patients receiving clinical care at the 9 IM clinics, which comprise the Bravewell Clinical Network and BraveNet PBRN. This effort provided important information about patients receiving care at these IM clinics as well as the type of medical condition and IM treatments patients were receiving.

## Methods

The current pilot study extends the prior study by collecting patient-reported outcomes every 6 weeks for 6 months from patients receiving various types of IM services (including acupuncture, Ayurveda, massage therapy, biofeedback, IM physician consults, and various mind/body practices). Patients are asked to complete demographics, substance use, exercise habits, Perceived Stress Scale, Arizona Integrated Health Outcomes, and Patient Related Outcomes Measurement Information System-29 (which measures physical function, anxiety depression, fatigue, sleep disturbance, social roles, and pain interference/intensity) measures. Questions examining the patients’ prior IM history, perceived benefits of IM treatments, and satisfaction with IM clinic are also included. Medical records are reviewed to obtain billing and healthcare utilization information. While electronic questionnaires are completed using the REDCap (Research Electronic Data Capture) system, patients may also complete the questionnaires via paper format.

## Results

The Penny George Institute for Health and Healing (Minneapolis, MN) has been successfully piloting this project and 200 participants are enrolled to date. Additional BraveNet PBRN sites are joining the pilot study and expanded results of the entire pilot study will be presented.

## Conclusion

Practice-based research and patient-reported outcome measures will be key factors in demonstrating the effectiveness of IM interventions in the treatment of many clinical conditions. Multicenter participation provides increased enrollment and allows for generalizability of results from which future randomized controlled trials may be developed.

